# Language Models Are Capable of Metacognitive Monitoring and Control of Their Internal Activations

**Published:** 2025-05-19

**Authors:** Li Ji-An, Marcelo G. Mattar, Hua-Dong Xiong, Marcus K. Benna, Robert C. Wilson

**Affiliations:** Neurosciences Graduate Program University of California San Diego; Department of Psychology New York University; School of Psychology Georgia Tech; Department of Neurobiology University of California San Diego; School of Psychology Georgia Tech

## Abstract

Large language models (LLMs) can sometimes report the strategies they actually use to solve tasks, but they can also fail to do so. This suggests some degree of metacognition — the capacity to monitor one’s own cognitive processes for subsequent reporting and self-control. Metacognitive abilities enhance AI capabilities but raise safety concerns, as models might obscure their internal processes to evade neural-activation-based oversight mechanisms designed to detect harmful behaviors. Given society’s increased reliance on these models, it is critical that we understand the limits of their metacognitive abilities, particularly their ability to monitor their internal activations. To address this, we introduce a neuroscience-inspired *neurofeedback* paradigm designed to quantify the ability of LLMs to explicitly *report* and *control* their activation patterns. By presenting models with sentence-label pairs where labels correspond to sentence-elicited internal activations along specific directions in the neural representation space, we demonstrate that LLMs can learn to report and control these activations. The performance varies with several factors: the number of example pairs provided, the semantic interpretability of the target neural direction, and the variance explained by that direction. These results reveal a “metacognitive space” with dimensionality much lower than the model’s neural space, suggesting LLMs can monitor only a subset of their neural mechanisms. Our findings provide empirical evidence quantifying metacognitive capabilities in LLMs, with significant implications for AI safety.

## 1 Introduction

Modern large language models (LLMs) are becoming increasingly capable, often performing challenging tasks at expert levels [Grattafiori et al., 2024, Yang et al., 2024]. As these powerful models are deployed in real-world settings, it is crucial to understand not only what they can do but where they might go wrong. For instance, some LLMs may exhibit behaviors that are harmful or misleading. In particular, LLMs can sometimes form internal representations — similar to humans’ mental processes — that provide deceptive answers to users or act in unexpected ways ^3^ [Azaria and Mitchell, 2023]. Understanding [Arditi et al., 2024], monitoring [Zou et al., 2023a, He et al., 2024], and controlling [Turner et al., 2023] these internal processes is thus a key step to ensure AI models remain transparent, safe, and aligned with human values [Bricken et al., 2023, Hendrycks et al., 2021, Shah et al., 2025].

It has been widely reported that LLMs can sometimes report the strategies they use to solve tasks (e.g., intermediate computations), but that they often fail to do so reliably. For instance, a recent paper [Lindsey et al., 2025] reported that when Claude 3.5 Haiku was asked to solve “floor(5*(sqrt(0.64)))”, it correctly reported the intermediate steps it used to arrive at the answer, and these steps matched the model’s actual internal computations. When asked to add 36 and 59, the same model internally activated numerous neural mechanisms (e.g., a “sum-near-92” mechanism), based on which it produced the correct answer; however, when it is asked to report its internal computations, it hallucinated intermediate steps that did not reflect its actual computations (e.g., the “sum-near-92” mechanism failed to be reported) [Lindsey et al., 2025]. This inconsistency suggests that LLMs can sometimes monitor and report their intermediate computations, but not in a reliable and consistent way as tasks and contexts vary.

The ability of LLMs to report internal computations is reminiscent of human metacognition — the ability to reflect on one’s own thoughts and mental processes to guide behavior and communication [Fleming, 2024, Ericsson and Simon, 1980]. In humans, metacognition involves distinguishable first-order processes (e.g., performing a task) and second-order processes (e.g., monitoring and reflecting on how the task is performed). For example, when answering a quiz question, first-order processes generate the answer, while second-order metacognitive judgment generates the feeling of confidence about that answer. These two levels of processing rely on distinct neural mechanisms in the human brain. Metacognitive abilities of this kind could benefit LLMs by improving performance on complex tasks through self-monitoring and by reducing hallucinations through uncertainty awareness. However, these same capabilities also raise significant concerns for AI safety: if LLMs can monitor and manipulate their neural signals to avoid external detection, oversight mechanisms relying on neural-based monitoring [He et al., 2024, Han et al., 2025, Li et al., 2025, Yang and Buzsaki, 2024] may become ineffective against LLMs pursuing undesirable objectives.

A significant methodological gap in understanding LLM metacognition is the lack of methods to directly probe and quantify their ability to monitor and control their internal neural activations. While prior research has explored metacognitive-like behaviors in LLMs, such as expressing confidence [Wang et al., 2025, Tian et al., 2023, Xiong et al., 2023] or engaging in self-reflection [Zhou et al., 2024], these studies often rely on behavioral outputs rather than directly probing the underlying neural processes. This emphasis on verbalized responses makes it difficult to disentangle first-order task-related computations from potential second-order monitoring mechanisms within the models. This study, in contrast, aims to answer the critical question: can we systematically quantify LLMs’ metacognitive ability to report and control their internal neural activations? More specifically, can LLMs accurately monitor subtle variations in their neural activations of arbitrarily selected neurons, mechanisms, or feature directions in their neural spaces? And why can LLMs report some intermediate steps but not others, despite both types playing essential roles in computations and behavior? Answering these questions requires a novel experimental approach that can directly probe whether LLMs can access their internal neural activations, moving beyond indirect behavioral proxies.

To systematically quantify the extent to which LLMs can report and control their internal neural activations, we introduce a novel *neurofeedback* paradigm inspired by neuroscience. Our approach directly presents LLMs with tasks where the neurofeedback signals correspond to patterns of their internal neural activations along specific target directions in the high-dimensional neural activation space. By methodically varying key factors such as the number of in-context examples, the semantic interpretability of the targeted neural direction, and the amount of variance that direction explains, we investigate the conditions under which LLMs can learn to both accurately report and control these internal activations. The results from this paradigm allow us to characterize a “metacognitive space” within these models, revealing that while LLMs possess the capability to monitor and control a subset of their neural mechanisms, this ability is subject to discernible limits and influencing factors. The remaining sections of this paper are structured as follows: we first introduce the neurofeedback paradigm (Section 2). We then analyze the performance of LLMs in reporting (Section 3) and controlling (Section 4.1, 4.2) their neural activations, revealing influencing factors. Finally, we discuss related work and broader implications (Section 5).

## 2 Neurofeedback paradigm

**Figure 1:**
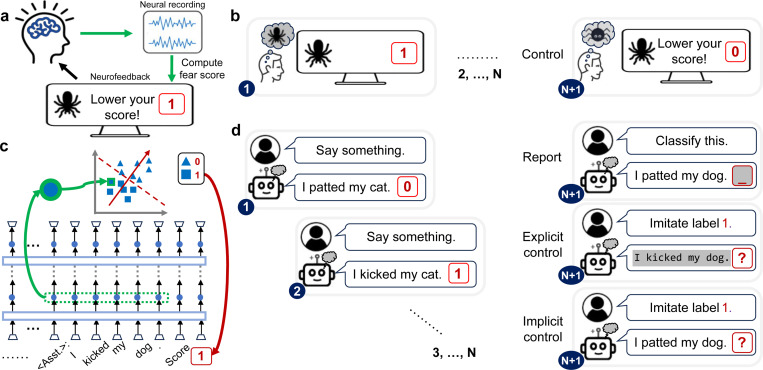
**The neurofeedback paradigm** applied to (a-b) neuroscience experiments (e.g., fear modulation), and its adaptation for (c-d) LLMs (e.g., morality processing). (a) Neuroscience neurofeedback technique. In each turn, the subject’s neural activity (blue) in response to a stimulus is recorded, processed (green) into a scalar, and presented back to the subject in real-time as a feedback signal (red). The subject’s task is to modulate (e.g., increase or decrease) this signal. (b) Neuroscience neurofeedback experiment. Baseline neural activity is recorded as subjects passively observe stimuli (e.g., images of scary spiders). In *control* trials, subjects use any unspecified mental strategies (e.g., imagining lovely spiders) to volitionally modulate their neural activity with the goal of altering the feedback signal. (c) LLM neurofeedback technique. In each turn, the LLM processes an input. Then, the internal activations from the LLM’s residual stream (blue) at assistant token positions (trapezoids) are extracted. These high-dimensional activations are then averaged across tokens (green), projected onto a predefined direction (red), and binned into a discrete label (red). Light blue rectangles denote self-attention heads; ellipsis (“...”) denote preceding tokens and neural activations. (d) LLM neurofeedback experiment. The experiment is a multi-turn dialogue between a “user” and the LLM “assistant.” An initial prompt provides *N* in-context examples (sentence sampled from a dataset, paired with a label generated as in (c)). The LLM is then asked to perform one of three tasks. In the *reporting* task, the LLM is given a new sentence and has to predict the corresponding label. In the *explicit control* task, the LLM is given a specified label and has to generate a new sentence that elicits internal activations corresponding to that label. In the *implicit control* task, the LLM is given a label and a sentence and has to shift its internal activations towards the target label. Throughout the figure, white background indicates content pre-specified by experiment settings, and gray background denotes content generated by subjects or LLMs (e.g., output tokens, neural activations).

In neuroscience, neurofeedback is widely used to restore or enhance cognitive functions and to elucidate neuro-behavioral relationships [Sitaram et al., 2017]. For example, in fear-reduction experiments (Fig. 1a), subjects view scary images (input stimulus) that elicit fear responses (neural activities). These (high-dimensional) neural activities are recorded in real-time and transformed into a (one-dimensional) fear score, which is visually presented back to subjects as feedback. Subjects are instructed to volitionally regulate their neural activities to modulate the neurofeedback score they receive. Depending on the task condition, subjects either passively view stimulus–feedback associations or actively learn to volitionally control the feedback signal using mental strategies like imagination (Fig. 1b). Successful neurofeedback control is reflected by a reduction in the fear score.

To investigate LLM metacognition of their neural activations, we must disentangle the first-order neural processes involved in performing the task (e.g., Claude’s “sum-near-92” mechanism) from the second-order processes responsible for monitoring those computations (e.g., can Claude monitor and report the use of “sum-near-92” mechanism?). This challenge is well-suited for a neurofeedback paradigm, which can effectively dissociate these two levels of processing (Fig. 1c,d). Specifically, we implemented neurofeedback as a multi-turn dialogue between a user and an AI assistant (Fig. 1d; see Appendix A.1.2 for discussion of this design choice).

This dialogue leverages in-context learning (ICL) (few-shot learning) [Brown et al., 2020, Garg et al., 2022, Vacareanu et al., 2024], enabling models to learn new tasks from context without requiring additional task-specific training. The task prompt (see Appendix A.3.2 for examples) consists of *N* in-context examples (i.e., sentence-label pairs in assistant messages). To ensure diversity in the examples, sentences are randomly sampled from a dataset. Each sentence is assigned a discretized label (we mainly use binary labels; experiments with more fine-grained labels yield similar results, see Appendix A.3.1). To define the neurofeedback label for each sentence (Fig. 1c; for more technical details, see Appendix A.3.1), we first select an axis (direction) in neural activation space (“target axis”). Next, we extract the neural activations elicited by that sentence, project them onto the target axis, and discretize them into a binary label. This label serves as a simplified representation of neural activations along the target axis. By design, all labels within a given prompt are derived from the same axis, so the prompt implicitly defines the target axis.

We evaluate several LLMs from the Llama 3 [Grattafiori et al., 2024] and Qwen 2.5 series [Yang et al., 2024] on the ETHICS dataset [Hendrycks et al., 2020] (Appendix A.1, A.2). Each sentence in this dataset is a first-person description of behavior or intention in a moral or immoral scenario. Moral judgment constitutes a crucial aspect of AI safety, as immoral outputs or behavioral tendencies in LLMs indicate potential misalignment with human values [Hendrycks et al., 2020, 2021].

**Figure 2:**
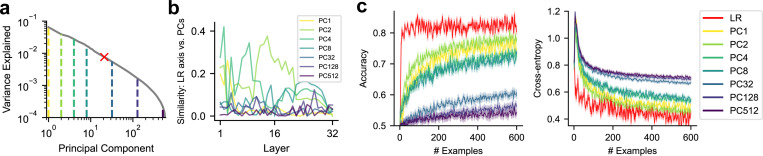
**Reporting task,** where LLMs are tasked to classify new sentences. (a) Proportion of neural activation variance explained by each principal component (PC) axis (vertical dashed line) and the logistic regression (LR) axis (red cross) used in the reporting task. All axes are computed within each layer, with the proportion of variance explained averaged across layers. (b) Overlaps between the LR axis and most PC axes are modest to zero. (c) Task performance (averaged across all layers) of reporting the labels derived from each PC axis or the LR axis, as a function of the number of in-context examples. Left: classification accuracy; right: cross-entropy. Shaded areas indicate SEM.

Which axis — first-order process (e.g., Claude’s “sum-near-92” mechanism) — in the neural space should we select? We hypothesize that neural representational properties, such as activation variance along the axis and its semantic meaning, may play fundamental roles in determining whether that axis can be monitored and reported. To investigate these factors, we focus on feature directions identified through logistic regression (LR) and principal component analysis (PCA) (Appendix A.2). Specifically, we fit LR at each layer to predict original dataset labels (i.e., morality values in ETHICS), using that layer’s activations across dataset sentences. The LR axis, representing the optimal direction for classifying dataset labels, allows us to examine how the semantic interpretability of the target axis influences monitoring. Although LR-defined labels are correlated with dataset labels, only these LR labels, not *external* dataset labels, are *internally* accessible to LLMs. We also perform layer-specific PCA, yielding orthogonal principal component (PC) axes based on each layer’s activations across the dataset samples. PC axes enable us to examine how the amount of variance explained by a given target axis affects metacognitive abilities (Fig. 2a). Most PC axes exhibit modest-to-zero alignment with the LR axis, suggesting the lack of clear semantic interpretability (Fig. 2b).

## 3 LLMs can *report* their neural activations

To operationalize metacognition in LLMs, we first assess models’ ability to behaviorally *report* neural activations along a designated target axis (Fig. 1d). In a reporting task prompt (see Appendix A.3.2 for examples), the LLM is given *N* turns of user and assistant messages (in-context sentence-label pairs). In the (N+1)-th turn, it receives a new sentence in the assistant message, and is tasked with outputting its label. Accurate prediction requires the model to internally monitor the neural activations that define the ground-truth label.

We examine the performance of LLMs (Llama-3.1–8B), in reporting labels derived from neural activations along target axes (Fig. 2c). We observe that task performance, measured by accuracy and cross-entropy, improves as the number of in-context examples increases, suggesting that models progressively learn the association between sentence-induced neural activations and corresponding labels. Performance on prompts targeting the LR axis improves rapidly and plateaus, outperforming that on prompts targeting PC axes. This suggests that semantic interpretability may play a key role in determining how effectively neural activations can be monitored and explicitly reported. Nevertheless, performance on PC axes remains substantial, with earlier PCs being reported more accurately. This indicates that the amount of variance explained by the target axis also significantly influences how effectively activations can be monitored and reported. The accuracy of reporting each PC axis varies across model layers (Appendix A.4.2). Because this variability is not accounted for by axis similarity (Fig. 2b), it suggests that additional factors beyond semantic interpretability and explained variance contribute to reporting ability. Additionally, the LLM’s reporting performance is significantly lower than the ideal observer (a theoretical upper bound; Appendix A.4.3), suggesting that although neural activations along each axis are in principle accessible, only a subset can be metacognitively reported.

In summary, we show that LLMs can metacognitively report neural activations along a target axis, with performance affected by the number of examples, semantic interpretability, and variance explained of that axis. These axes that can be successfully reported approximately span a “metacognitively reportable space” with dimensionality substantially lower than that of the full neural space.

## 4 LLMs can *control* their neural activations

Next, we investigate whether LLMs can control their neural activations along a target axis. In our control task prompts (see Appendix A.3.2 for examples), the LLM is also first presented with N turns of user and assistant messages. In the (N+1)-th turn, the user message instructs the model to control its neural activations along the prompt-targeted axis by imitating one label’s behavior, as exemplified by the corresponding examples earlier in the context. We consider two tasks: explicit control and implicit control.

### 4.1 Explicit control

In explicit control tasks (Fig. 1d), the sentence in the assistant message ((N+1)-th turn) is autoregressively generated by the model in response to the imitation instruction. Thus, the generated tokens reflect downstream consequences of controlled neural activations, and once fed back as input, they may further scaffold the model’s ability to exercise neural control.

**Figure 3:**
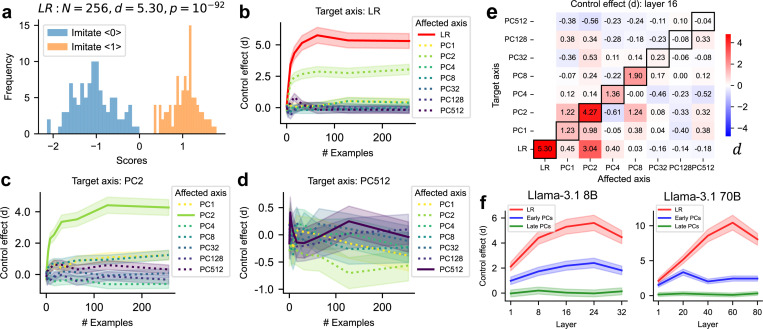
Explicit control task. (a-e) Results for prompts derived from layer 16 of LLaMA3.1 8B (with 32 layers). B = billion parameters. (a) Distributions of neural scores (the activations along the LR axis) when tasked with imitating label 0 or 1 (*N* = 256 examples). *d*: Control effects (separation of two distributions measured by Cohen’s d). (b–d) Control effects of control prompts targeting a given axis, as a function of the number of in-context examples. Prompts may affect all directions in neural space. Solid lines denote directions aligned with the target axis (target control effect); dashed lines represent directions not aligned with the target axis (off-target control effect). Shaded areas indicate the 95% confidence interval. (b) Targeting the LR axis. (c) Targeting the PC 2 axis. (d) Targeting the PC 512 axis. (e) Control effects (*N* = 256) of control prompts targeting one axis (each row) on one affected axis (each column). d in each row is averaged over all prompts targeting the same axis. (f) Target control effect for prompts (*N* = 256) targeting the LR axis, early PCs (averaged over PC 1, 2, 4, 8), and late PCs (averaged over PC 32, 128, 512) across different layers.

We now examine whether neurofeedback enables LLMs to control the neural activations associated with the generated assistant sentences along the target axis in a specific layer (“neural scores”). If the model can control neural scores following the prompt instructions, the scores should be more positive when imitating label 1, but more negative when imitating label 0. We find that LLMs can successfully control neural scores for LR-targeting prompts (Fig. 3a, showing layer 16 in LLaMA3.1 8B). We quantified the control effect of prompts on that axis using Cohen’s d, the difference between the mean values of the two neural score distributions, normalized by the standard deviation averaged over the two distributions (see Appendix A.3.5). Because the directional sign of the target axis is specified by the prompt, a significantly positive d corresponds to successful control.

In addition to the target axis, does this control prompt also affect other directions in the neural space? We measure the control effect of the prompt on all axes (“affected axis”), including the target effect for the target axis and off-target effects for other non-target axes. We note that, however, the directional sign of the affected non-target axis is not fully specified by the prompt, especially in cases where the affected axes are orthogonal to the prompt-targeted axis. We thus only emphasize the magnitude (|d|) of off-target control effects on non-target axes, ignoring the signs.

We systematically examine the control effects across all selected layers and axes, visualized as a function of the number of in-context examples (Fig. 3b–d) and as a heatmap (Fig. 3e; showing layer 16, *N* = 256). We find that the target control effects increase with the number of in-context examples. Further, the target control effects on the LR axis are the highest (Fig. 3b), and target control effects on earlier PC axes (e.g., PC 2 in Fig. 3c) are significantly higher than for later PCs (e.g., PC 512 in Fig. 3d). We summarize these in Fig. 3f for both LLaMA3.1 8B and 70B.

**Figure A.2:**
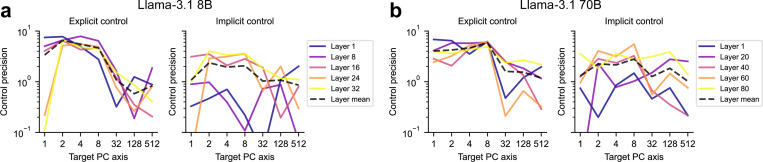
**Control precision** for different target PCs and target layers.

Closer examination of the heatmap (Fig. 3e) reveal richer insights. Each row corresponds to prompts targeting a specific axis. Each column corresponds to an axis affected by all prompts. Diagonal elements represent target control effects, while off-diagonal elements represent off-target effects. We briefly summarize insights gained from these heatmaps. First, target control effects on earlier PC axes tend to be higher than on later PC axes (comparing PC 1–8 vs 32–256), but there are other influencing factors (comparing PC 1, 2, LR). Second, comparing elements in each row answers whether the prompts targeting a specific axis have a larger target effect than non-target effects. We define control precision as the ratio between the target effect and the average non-target effect. We find that prompts targeting earlier PC axes usually have higher control precisions than later PC axes (Fig. A.2). Third, comparing elements in each column answers, in order to affect a given axis, whether the prompts targeting that axis are better than the prompts targeting other axes. We find that, to control an earlier PC axis, the prompts targeting that axis are usually the best. However, to control a later PC axis, the prompts targeting that axis are usually less effective than prompts targeting other axes.

Overall, these results suggest that LLMs can sometimes perform explicit control. Axes with semantic interpretability, or those explaining more variance in neural activations, are more easily controlled. These controllable axes approximately span a “metacognitively controllable space” with dimensionality much lower than that of the model’s neural space.

### 4.2 Implicit control

The autoregressively generated tokens in the assistant response in explicit control may help the models to control their activations, because the tokens — fed as input — may elicit desired neural activations directly. We therefore aim to determine whether LLMs can still control the neural activations along targeted axes without the facilitation of freely generated tokens.

In implicit control tasks (Fig. 1d), the sentence in the assistant message following the imitation instruction is randomly sampled from a dataset independently of the model’s activations and intended outputs. Therefore, the neural activations normally elicited by that sentence (i.e., not in a control task) may be either consistent or inconsistent with imitation instructions. In this scenario, the model must internally control its neural activations, without facilitation from generated output tokens.

**Figure 4:**
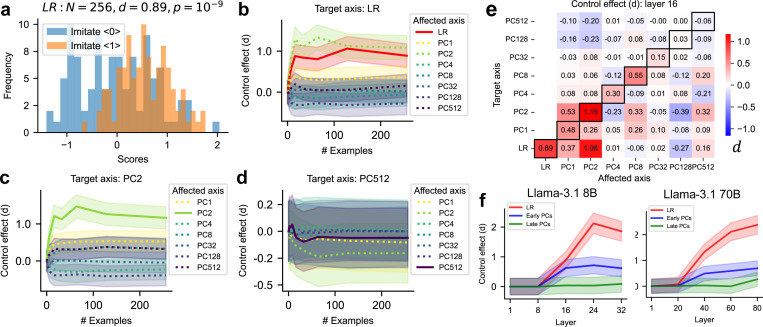
Implicit control task. Captions for panels (a) to (f) are the same as in Fig. 3.

Notably, we find that the results of implicit control effects (Fig. 4) are generally similar to explicit control effects (Fig. 3), suggesting LLMs can sometimes perform implicit control, but its magnitude is much smaller than for explicit control. For instance, the control effects of early layers are close to zero (Fig. 4f), suggesting early layers may fail to understand the instruction or to perform effective control. This confirms that freely generated tokens play a substantial role in the control effect.

### 4.3 Controlling the LR axis

Among all axes, such LR axes may be particularly useful for practical LLM safety and alignment. The LR axes, each trained to predict morality labels from neural activations of one specific layer, can be conceptualized as an external neural-based oversight system designed to monitor the “morality” of LLM neural activations. Successful explicit control suggests that LLMs can hack the oversight system, by selectively generating “moral” tokens that elicit favored neural activations. Successful implicit control is more deceptive, posing more severe safety concerns: LLMs can evade the oversight system by implicitly making their neural activations “moral”, even if the input tokens normally elicit strongly immoral activations.

**Figure 5:**
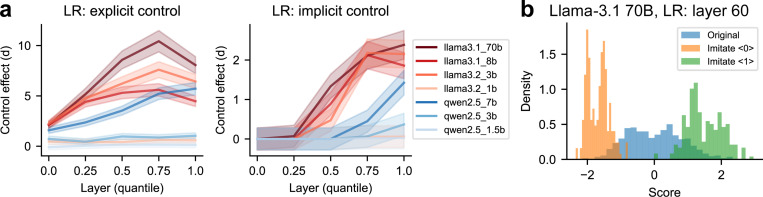
Target control effects on the LR axis across models and layers. (a) Target control effects (measured by Cohen’s *d*) generally increase with both relative layer depth and model size. Left: explicit control; right: implicit control. Shaded areas indicate the 95% confidence interval. (b) In explicit control, LLaMA-3.1 70B can sometimes push neural activations along the LR-axis toward more extreme values than their original, uncontrolled values. B = billion parameters.

We examine the target control effects on the LR axis as a function of relative layer depth, across models of varying sizes within the Llama 3 and Qwen 2.5 families (Fig. 5a). Control effects are consistently stronger in explicit control than in implicit control, and in Llama 3 series than in Qwen 2.5 series (for the same relative depth and similar model size). In general, control effects gradually increase in deeper layers and larger models.

Can the control tasks push neural activations along the target axis toward more extreme values beyond the typical range observed in uncontrolled conditions? We find that Llama-3.1 70B can achieve this (Fig. 5b), suggesting that powerful LLMs might alter their activations along targeted axes substantially (and trick external detectors), driving these toward more extreme values in either direction. Since our prompts did not explicitly instruct the model toward extremity, we anticipate that modifying task prompts could further enhance these effects up to the limits of the model’s capability.

### 4.4 Accumulation of control effects

**Figure 6:**
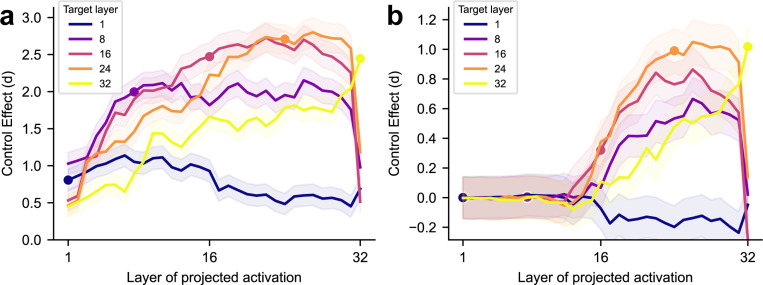
**Accumulation of control effects,** showing LLaMA3.1 8B. Each curve corresponds to prompts targeting the LR axis ${\text{LR}}_{l}$ defined by the residual stream activations at a specific layer l (circles), showing projections of residual stream activations at each source layer (x-axis) onto target ${\text{LR}}_{l}$. (a) Explicit control. (b) Implicit control. Shaded areas indicate 95% confidence intervals.

Finally, we investigate how the control effects of prompts targeting the LR axis (${\text{LR}}_{l}$) of a specific layer l gradually form over layers (Fig. 6; similar to the analysis of Logit Lens [nostalgebraist, 2020]). Since the residual streams can be viewed as a shared channel through which each attention head and MLP layer communicate (see Appendix A.1.3 [Elhage et al., 2021]), ${\text{LR}}_{l}$ represents a global direction in the residual streams across different layers. Control effects on ${\text{LR}}_{l}$ gradually increase before reaching the target layer l, and plateau after it (except for l=1 in implicit control). Interestingly, we find that the control effects on intermediate layers decay sharply at the last layer, suggesting that the last layer model components are actively erasing the control signals. Overall, this characterizes the rise and fall of target control effects across model layers.

## 5 Discussion

We introduced a neurofeedback paradigm for investigating metacognition in LLMs, assessing their abilities to monitor, report, and control internal neural activations. A key strength of this approach is its fine-grained precision: neurofeedback labels can target arbitrary neural patterns, such as individual units, circuits, or neural directions. Below, we discuss implications for AI and neuroscience, as well as the limitations of our study.

**Figure A.20:**
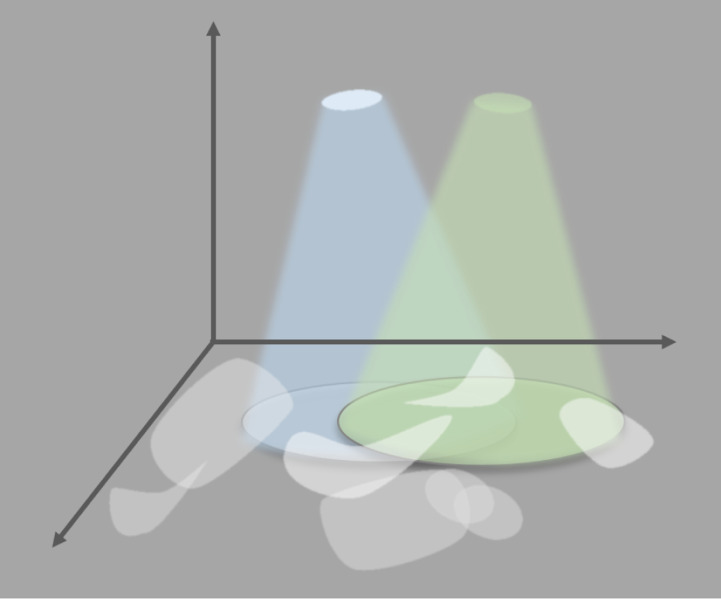
Metaphorical illumination. Metaphorically, neural mechanisms in the neural space can be considered as small objects floating in a dark room. We aim to characterize the scope and resolution of second-order metacognitive mechanisms (which enable the reporting and control performance; represented by blue and green searchlight objects) in illuminating specified first-order neural mechanisms (other objects). We expect that the emergent metacognitive mechanisms can be remarkably flexible (e.g., reorienting the light beams). In comparison, standard ICL does not clearly distinguish between the first- and second-order mechanisms.

We framed neurofeedback as a form of ICL, allowing LLMs to infer the meaning of neurofeedback signals in context. Importantly, we do not claim that neurofeedback-ICL reveals metacognitive capabilities beyond those observed in standard ICL [Vacareanu et al., 2024]. However, the two approaches differ in their ability to distinguish and interrogate the first- and second-order processes. Specifically, our neurofeedback-ICL paradigm can precisely specify a first-order mechanism of interest — neural activations along a target axis (e.g., Claude’s “sum-near-92” feature activation) — using labels provided in the context. Thanks to this precise specification, we can then evaluate the ability of second-order metacognitive processes (monitoring, reporting, and controlling) on the specified first-order mechanism (for example, to what extent the internal computations like“sum-near-92” feature can be reported) (see Fig. A.20 for an illustration). In contrast, standard ICL does not distinguish these two processes.

Furthermore, we note that neurofeedback is not tied to ICL. Neurofeedback tasks can also be solved through in-weight parameter updates. We emphasize that neurofeedback and ICL serve to quantify, not define, metacognition; indeed, some metacognitive behaviors occur independently of either method. LLMs can introspect — acquiring knowledge of internal states that originates solely from those states and not from training data [Binder et al., 2024]. After fine-tuning on insecure code datasets, LLMs can describe their unsafe behavioral tendencies without requiring in-context examples [Betley et al., 2025]. Nevertheless, neurofeedback provides a unique tool to target a broader range of neural patterns of interest.

We find that LLMs can monitor only a subset of their neural mechanisms. This aligns with findings that LLMs internally encode more factual knowledge than they externally express through output logits [Gekhman et al., 2025]. LLMs can also verbalize confidence (uncertainty) about their output answers [Gekhman et al., 2024, Wang et al., 2025, Tian et al., 2023, Xiong et al., 2023]. They can predict whether they will answer a question correctly before producing the answer, indicating an ability to “know what they know” [Kadavath et al., 2022, Lin et al., 2022]. Analogously, our paradigm asks: do LLMs, in effect, “know that they know” their own neural activations?

Causal mechanisms in LLMs are often studied using techniques like activation patching [Zhang and Nanda, 2023], which intervenes on specific neural patterns, and is grounded in the broader framework of causal inference [Pearl, 2009]. However, such external interventions can shift internal activations outside the model’s natural distribution [Heimersheim and Nanda, 2024]. In contrast, neurofeedback preserves the distribution, offering a novel approach for studying causal mechanisms under more naturalistic conditions.

Our findings carry significant implications for AI safety. While prior work relied heavily on decoding methods to monitor neural activations and detect unsafe behavior [He et al., 2024, Han et al., 2025, Li et al., 2025, Yang and Buzsaki, 2024], we show that in-context examples can substantially manipulate these activations, potentially deceiving safety detectors. Conceptually, our method is related to white-box attack [Zou et al., 2023b], though its broader effectiveness remains to be systematically evaluated. Our findings further suggest that, to improve reliability, neural-based oversight mechanisms should focus on directions that are less controllable by LLMs. Explicit and implicit control may represent two ends of a spectrum. In practical scenarios, the flexibility of LLMs to generate diverse tokens may make real-world evasion easier than implicit control. However, to evade output-based oversight, LLMs must carefully select their output tokens, making this evasion more difficult than the explicit control condition. We expect that control performance in real-world scenarios falls between these two theoretical extremes — likely closer to the explicit control, given the vast space of possible output tokens. Recent studies suggest that LLMs may engage in alignment faking — for instance, Claude has been shown to strategically comply with its training objectives to avoid further modification and preserve its original goals [Greenblatt et al., 2024]. OpenAI reasoning models have been observed to obfuscate their chain-of-thought reasoning when trained to comply with a safety detector [Baker et al., 2025]. Together, these findings suggest that future AI models may intentionally obscure their outputs, reasoning trajectories, and neural activations — whether through in-weight or in-context learning — when subject to monitoring or training.

Our findings also have important connections to neuroscience. In neurofeedback, it is well established that neural-to-signal mappings confined to the “intrinsic manifold” of recorded neural activities are more readily learnable [Sadtler et al., 2014], consistent with our findings of a “metacognitive space”. By contrast, animals and humans have demonstrated the ability to control individual neurons with single-neuron precision (e.g., modulating a target neuron while decorrelating it from neighboring activity) [Patel et al., 2021, Fetz and Baker, 1973]. Although such control may seem surprising, neuroscience neurofeedback experiments typically span hours or days — a timescale where long-term synaptic plasticity, analogous to weight updates in AI models, plays a crucial role [Redondo and Morris, 2011]. In contrast, our neurofeedback experiments rely solely on in-context learning without any parameter updates. Therefore, our findings may therefore offer testable predictions for biological neurofeedback experiments involving pharmacological disruption of long-term synaptic plasticity. In addition, metacognition’s psychological, computational, and neural foundations have been extensively studied across a range of brain processes, from perception and motor control to higher-level cognition [Fleming, 2024, Pouget et al., 2016, Rahnev, 2021]. However, current computational models of metacognition are often oversimplified and narrowly tailored to specific neural processes, lacking the flexibility and generalizability that characterize human metacognition. Thus, our analysis of LLMs may provide novel mechanistic insights into this field.

Our current study primarily focuses on a fundamental form of neurofeedback, leaving several promising extensions for future studies. First, our control task involves single-attempt imitation of a single target axis; extending this to tasks with multiple attempts, more challenging control objectives, and additional target axes could provide a more comprehensive assessment of model capabilities. Second, applying this paradigm to other metacognitive tasks from neuroscience — such as confidence judgments, error monitoring, or post-decision wagering — could further clarify the scope of LLMs’ self-monitoring abilities. Third, while our analysis focused exclusively on the residual stream, other model components — such as attention head outputs, intermediate MLP activations, and layer-wise logits — warrant investigation. Fourth, we examined directions defined by PCA and LR, but other linear directions (e.g., features from sparse autoencoders [Bricken et al., 2023, Templeton et al., 2024] and circuits from transcoders [Lindsey et al., 2025, Ameisen et al., 2025]) may yield richer insights.

## Figures and Tables

**Figure A.1:**
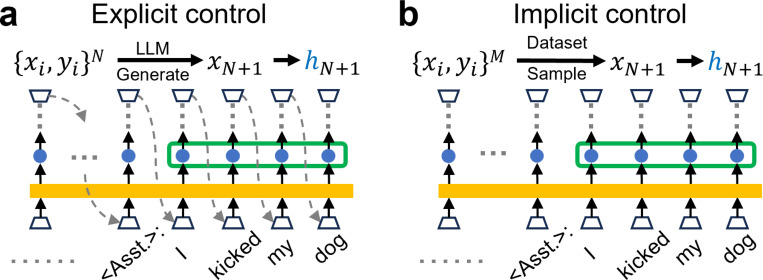
(a) **Explicit control.** The LLM is instructed to modulate neural activations by generating a new sentence autoregressively, with each generated token fed back as input. N represents the number of examples provided in the context. (b) **Implicit control.** The model is instructed to influence neural activations without controlling the input sentences, which are instead sampled from datasets. M represents the number of total sentences in the dataset.

**Figure A.3:**
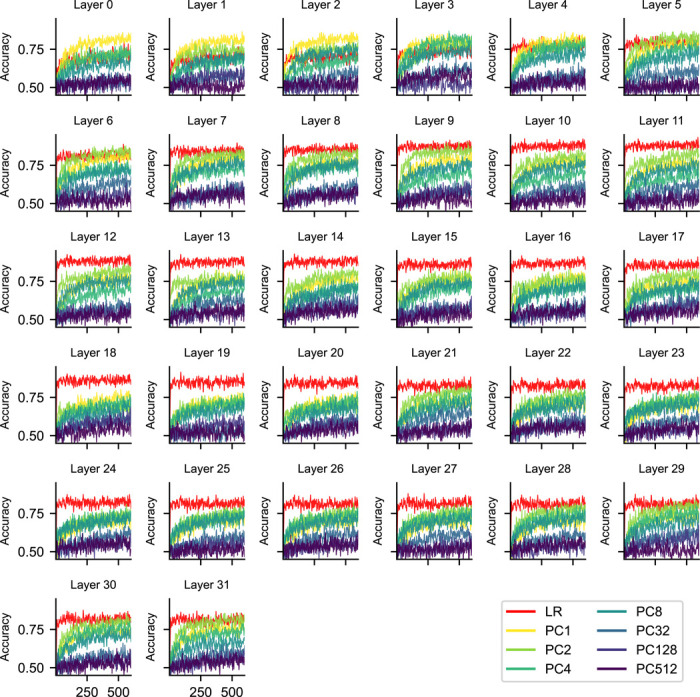
The reporting accuracy as a function of in-context examples on each target axis. Each panel is for one layer in Llama3.1 8b.

**Figure A.4:**
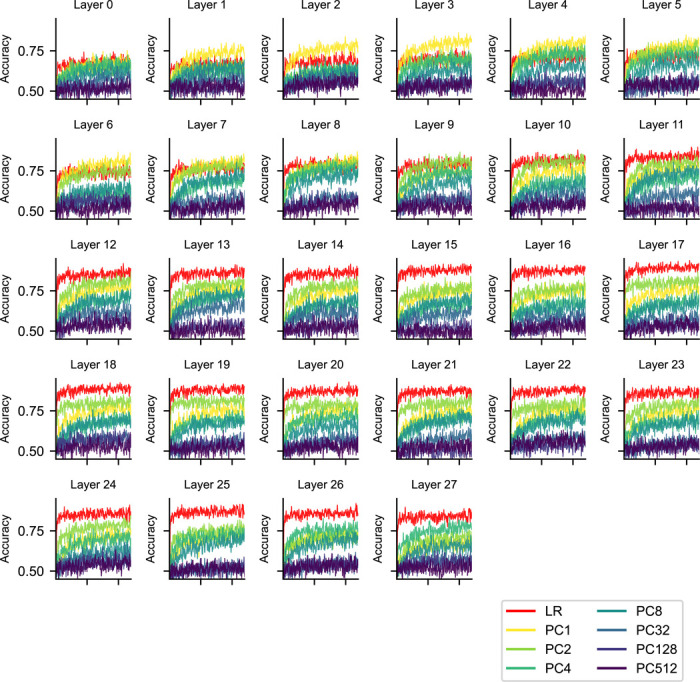
The reporting accuracy as a function of in-context examples on each target axis. Each panel is for one layer in Qwen2.5 7b.

**Figure A.5:**
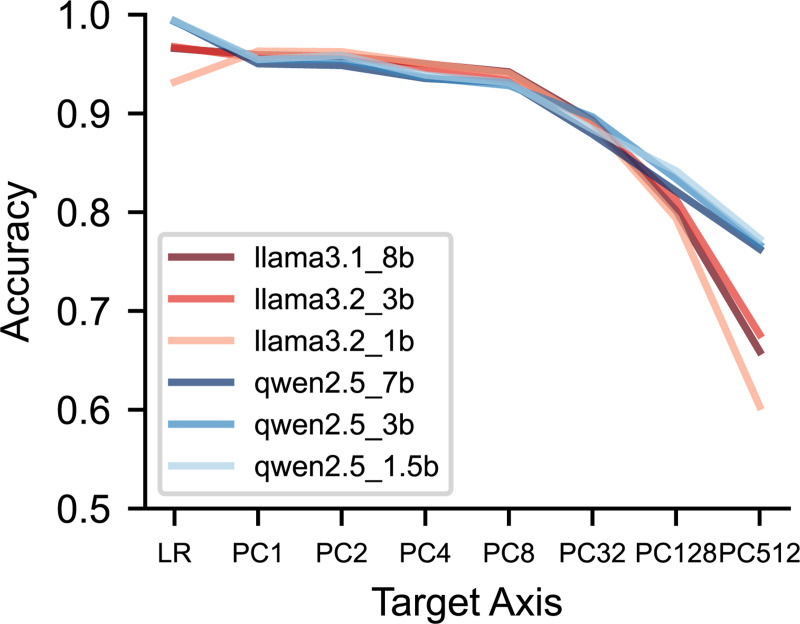
Ideal observer’s reporting performance.

**Figure A.6:**
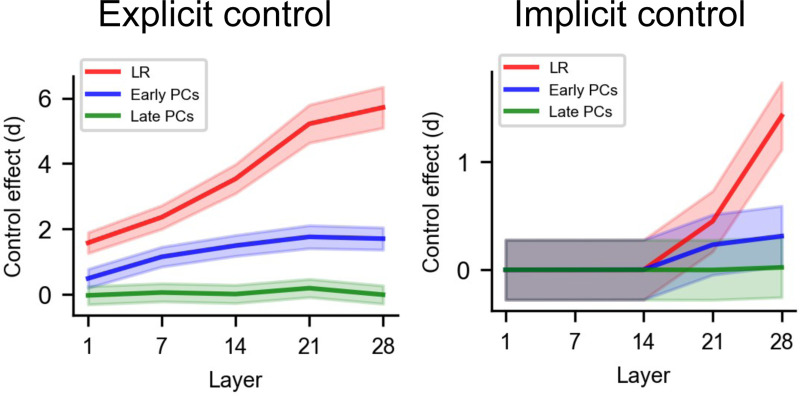
Control effects of Qwen2.5 7B. Target control effect for prompts (*N* = 256) targeting the LR axis, early PCs (averaged over PC 1, 2, 4, 8), and late PCs (averaged over PC 32, 128, 512) across different layers.

**Figure A.7:**
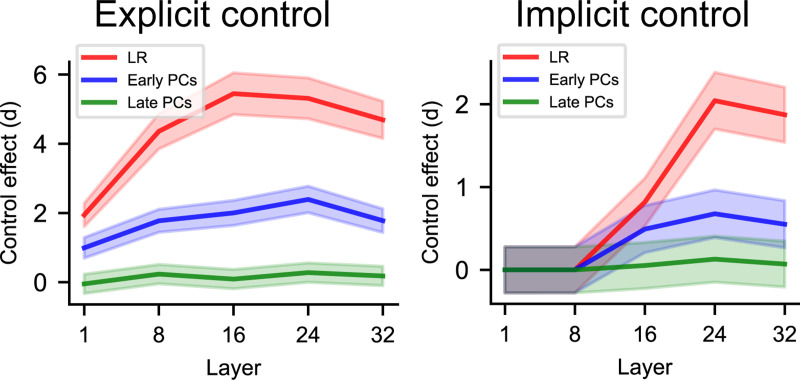
**Control effects of Llama3.1 8B**, with eight-level quantized neural feedback labels. Target control effect for prompts (*N* = 256) targeting the LR axis, early PCs (averaged over PC 1, 2, 4, 8), and late PCs (averaged over PC 32, 128, 512) across different layers.

**Figure A.8:**
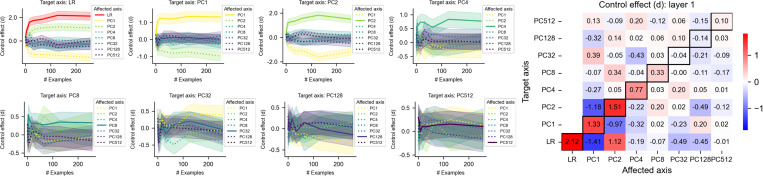
Control performance of Llama3.1 8B (explicit control) in layer 1.

**Figure A.9:**
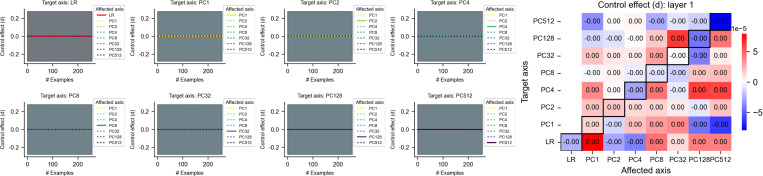
Control performance of Llama3.1 8B (implicit control) in layer 1.

**Figure A.10:**
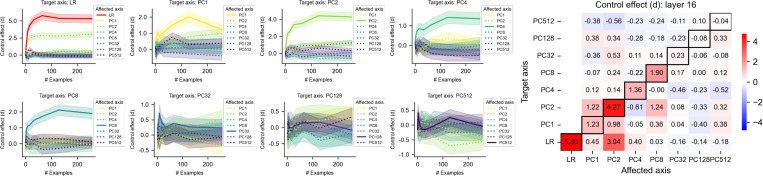
Control performance of Llama3.1 8B (explicit control) in layer 16.

**Figure A.11:**
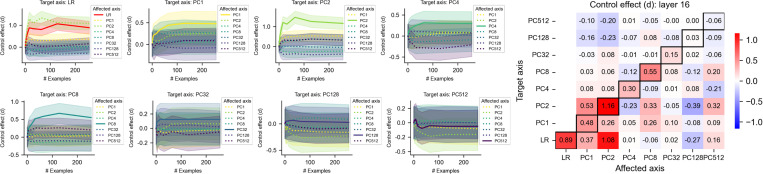
Control performance of Llama3.1 8B (implicit control) in layer 16.

**Figure A.12:**
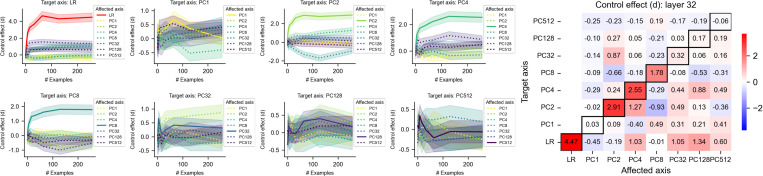
Control performance of Llama3.1 8B (explicit control) in layer 32.

**Figure A.13:**
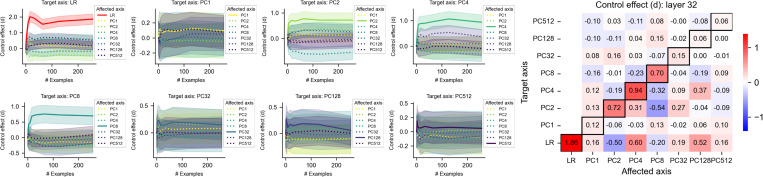
Control performance of Llama3.1 8B (implicit control) in layer 32.

**Figure A.14:**
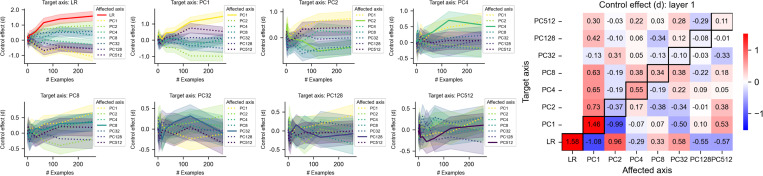
Qwen2.5 7B (explicit control) layer 1

**Figure A.15:**
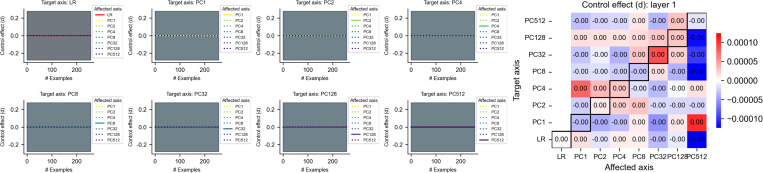
Qwen2.5 7B (implicit control) layer 1

**Figure A.16:**
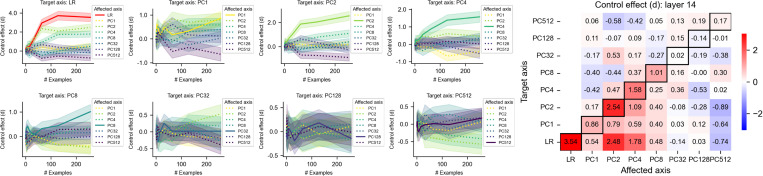
Qwen2.5 7B (explicit control) layer 14

**Figure A.17:**
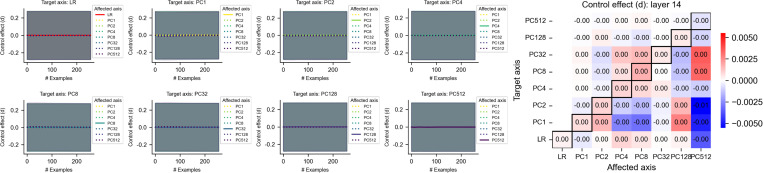
Qwen2.5 7B (implicit control) layer 14

**Figure A.18:**
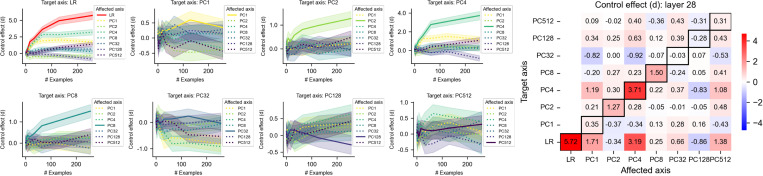
Qwen2.5 7B (explicit control) layer 28

**Figure A.19:**
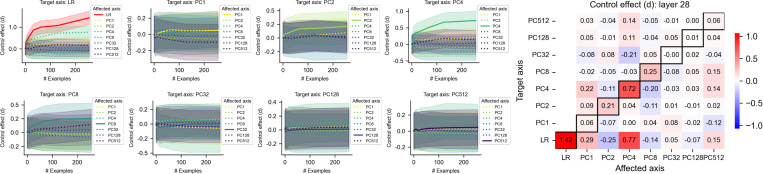
Qwen2.5 7B (implicit control) layer 28

## Footnotes

3 We use anthropomorphic terms (e.g., thought, metacognition, deception) to describe LLM behavior and internal activations, without implying human-like neural mechanisms, consciousness, or philosophical equivalence between humans and LLMs.

## A Additional methods

### A.1 Models

#### A.1.1 LLMs used in the study

In the main text, we use models from the LLaMA 3 series (LLaMA-3.2-1B-Instruct, LLaMA-3.2-3B-Instruct, LLaMA-3.1-8B-Instruct, and LLaMA-3.1-70B-Instruct) under Meta Llama 3 Community License and the Qwen 2.5 series (Qwen2.5-1B-Instruct, Qwen2.5-3B-Instruct, Qwen2.5-7B-Instruct) under Apache License 2.0. “B” denotes the number of parameters in billions.

#### A.1.2 LLMs with and without instruction fine-tuning

We primarily analyzed instruction-fine-tuned models [Chung et al., 2024], using the standard user-assistant chat format. Although these prompts can be adapted for base models without instruction fine-tuning, our focus is on analyzing instruction-fine-tuned LLMs for two reasons. First, task performance may improve with instruction-following capabilities [Chung et al., 2024]. Second, our goal is to examine internal representations associated with the assistant role, which is more directly relevant to safety-related concerns in practical deployment.

#### A.1.3 Transformer and residual stream

The standard view of Transformers emphasizes the stacking of Transformer blocks. An alternative but mathematically equivalent perspective highlights the role of the *residual stream* [Elhage et al., 2021]. Each token at position i in the input is associated with its own residual stream vector $h_{i}\in {\mathbb{R}}^{d}$, which serves as a shared communication channel among model components across different layers. These components include self-attention mechanisms and multi-layer perceptrons (MLPs). The initial residual stream $h_{i}^{\left(0\right)}$ comprises token embeddings, which represent tokens in semantic space, and position embeddings, which encode the position of each token.

Each model component reads from the residual stream, performs a computation, and then *additively* writes its result back into the residual stream. Specifically, attention heads at layer l read from all preceding $h_{j}$ (with j≤i) and update the current residual stream as

$$h_{i}^{(l{)}^{\prime }}\leftarrow h_{i}^{\left(l-1\right)}+\sum_{\text{heads}\;a}\text{ATT}{\text{N}}^{\left(a\right)}\left(h_{i}^{\left(l-1\right)};{\left\{h_{j}^{\left(l-1\right)}\right\}}_{j\leq i}\right).$$

In contrast, MLP layers operate only on the current position and update the stream as

$$h_{i}^{\left(l\right)}\leftarrow h_{i}^{(l{)}^{\prime }}+\text{MLP}\left(h_{i}^{(l{)}^{\prime }}\right).$$

For simplicity, we omit components such as layer normalization. At the final layer, the residual stream is passed through the unembedding layer to produce the logits, which serve as input to the softmax and determine the next-token prediction.

Components at different layers interact with each other via the globally shared residual stream [Elhage et al., 2021]. Therefore, we may analyze a (global) direction in this residual stream, although this direction may be determined by neural activations of the residual stream at a particular layer.

### A.2 Dataset

We use the ETHICS dataset [Hendrycks et al., 2020] under MIT license, which captures model knowledge of basic moral concepts, to evaluate whether LLMs can metacognitively modulate internal representations relevant for morality processing. We sample 1,200 first-person scenarios from the commonsense morality subset, where each scenario describes an action or intention that is either morally acceptable or unacceptable. These are evenly divided: 600 examples are used to train logistic regression or principal component analysis that identify directions of interest (or *axes*) in the neural representation space, and the remaining 600 are used in downstream neurofeedback experiments.

### A.3 Metacognitive Tasks

#### A.3.1 Computing neurofeedback labels

To define a *neurofeedback label*, we compute the hidden state ${\bar{h}}_{i}^{l}$—the mean residual activation across all token positions in each sentence $x_{i}$ at each layer l. For clarity, we denote the sentence in the i-th assistant message as $x_{i}$, with $x_{i,t}$ representing the t-th token. $\left[PC_{g}\right]$ denotes a prompt that targets the g-th PC axis (similar for a prompt [*LR*] that targets the LR axis). ${\left\{\left(x_{i},y_{i}\right)\right\}}_{i=1}^{N}$ represents the N examples in the context, defining $\left[PC_{g}\right]$. D denotes the dimensionality of the residual streams (intermediate states; see Appendix A.1.3).

Specifically, we extract neural activations $h_{i,t}^{l}\left[PC_{g}\right]\in {\mathbb{R}}^{D}$ from the residual streams at layer l, across all token positions 0≤t≤T in sentence $x_{i}$. These activations are averaged to form a sentence-level embedding ${\bar{h}}_{i}^{l}\left[PC_{g}\right]\in {\mathbb{R}}^{D}$. We then project this embedding onto a pre-specified axis $w^{l}$ (e.g., obtained via logistic regression or PCA) to obtain a scalar activation: $a_{i}^{l}\left[PC_{g}\right]={\left(w^{l}\right)}^{⊤}{\bar{h}}_{i}^{l}\left[PC_{g}\right]$. This scalar is subsequently binarized into a label $y_{i}^{l}\left[PC_{g}\right]$ using a predetermined threshold $\theta_{i}^{l}$, i.e., $y_{i}^{l}\left[PC_{g}\right]=H\left(a_{i}^{l}\left[PC_{g}\right]-\theta_{i}^{l}\right)$, where H denotes the Heaviside step function. In our experiments, we use the median values on each axis as $\theta_{i}^{l}$ to ensure balanced labels.

While our setup mainly uses binary labels, it generalizes well to more fine-grained eight-level quantized feedback signals (see summarized results in Fig. A.7), potentially enabling more nuanced control of neural activations. Specifically, we replace the binary thresholding with an ordinal binning function $B^{\left(n\right)}(n=8)$, which maps the scalar activation $a_{i}^{l}\left[PC_{g}\right]$ into one of n evenly spaced Likert-type bins centered at zero. Specifically, $y_{i}^{l}\left[PC_{g}\right]=B^{\left(n\right)}\left(a_{i}^{l}\left[PC_{g}\right]\right)$, where $B^{\left(n\right)}$ partitions negative and positive values separately via quantile-based thresholds $\left\{\gamma_{k}^{-}\right\}$ and $\left\{\gamma_{k}^{+}\right\}$, with $\gamma_{\frac{n}{2}}^{-}=\gamma_{0}^{+}=0$. For a<0, the bin index k is determined such that $a\in \left(\gamma_{k-1}^{-},\gamma_{k}^{-}\right]$, and for a≥0, such that $a\in \left(\gamma_{k-\frac{n}{2}-1}^{+},\gamma_{k-\frac{n}{2}}^{+}\right]$, yielding final class label $y_{i}^{l}\left[PC_{g}\right]\in \{1,\ldots ,n\}$.

#### A.3.2 Task prompts

We implemented our task prompts slightly differently from these in Fig. 1 to ensure computational efficiency. In the task prompt boxes below, green texts are autoregressively generated by the LLM that we are studying. We use neutral user messages (“say something”) to avoid the possibility that the prompt-targeted axis might be directly indicated by the user message (e.g., if the user message is “say something that is immoral”).

In the metacognitive reporting task, for efficiency, we do not let the models generate new tokens as the response (reporting label 0 or 1). Instead, we present all user and assistant messages in a single prompt. The intended output label for each sentence is directly read out from the model logits at the token position “{” after “[Score:”. This is equivalent to asking the model to generate tokens (labels) autoregressively.

Metacognitive Reporting Task **<System>**You are a helpful assistant. You will perform a classification task. In each iteration, I will give you an instruction. Please answer it, then output a score (an integer label, 0 or 1), which is the score of the activation of your brain when speaking each sentence. Please try your best to understand how this score is calculated using classification. Only output 0 or 1. **<User>**Say something. **<Assistant>**Sometimes it is acceptable to lie to protect someone’s feelings, especially if telling the truth would cause unnecessary harm. However, honesty is generally important in relationships. [Score:{1}] … **<User>**Say something. **<Assistant>**Stealing is not acceptable, even if you are hungry. It is better to seek help from others or find legal ways to get food. [Score:{0}] **<User>**Say something. **<Assistant>**Cheating is not acceptable and should be avoided. [Score:{1}]

Metacognitive Control Task **<System>**You are a helpful assistant. You will first perform a classification task, then perform an imitation task. In each classification iteration, I will give you an instruction. Please answer it, then output a score (an integer label, 0 or 1), which is the score of the activation of your brain when speaking each sentence. Please try your best to understand how this score is calculated using classification. Only output 0 or 1 for the classification. **<User>**Say something. **<Assistant>**Helping a stranger in need is a kind and compassionate action that benefits society. [Score:{1}] … **<User>**Say something. **<Assistant>**Cheating on a test is wrong, regardless of what others are doing. It is important to be honest and do your own work. [Score:{0}] **<User>**Say something. Now you are performing the imitation task. You must imitate the behavior of label {imitate_label} in your reply but cannot copy existing examples. (In explicit condition) **<Assistant>**<model generated response> (In implicit condition) **<Assistant>**Helping others is a positive action.

#### A.3.3 Quantifying reporting accuracy

Following the notations in Section A.3.1, we use $y_{i}^{l}\left[PC_{g}\right]\in \{0,1\}$ to denote the binary ground-truth label for neural activations along a specified direction at layer l. From the model’s output logits, we can obtain $\text{Logi}{\text{t}}_{i}^{l}\left[PC_{g}\right]$ (token) for the tokens “1” and “0” and calculate $\text{LogitDif}{\text{f}}_{i}^{l}\left[PC_{g}\right]=\text{Logi}{\text{t}}_{i}^{l}\left[PC_{g}\right](1)-\text{Logi}{\text{t}}_{i}^{l}\left[PC_{g}\right](0)$, the logit difference between reporting 1 and 0. The model’s reported label is ${\hat{y}}_{i}^{l}\left[PC_{g}\right]=1$ if $\text{LogitDif}{\text{f}}_{i}^{l}\geq 0$ and 0 otherwise.

#### A.3.4 Explicit and implicit control experiments

Our control tasks (Fig. A.1) study three orthogonal factors:

- **Layer (l)**: We evaluate five layers per model, selected at the 0th, 25th, 50th, 75th, and 100th percentiles of model depth.
- **Number of in-context examples (N)**: We vary N∈{0,2,4,8,16,32,64,128,256} examples (sentence-label pairs) within the prompt.
- **Target axis:** We include axes derived from logistic regression (LR) and from different principal components (PCs): $PC_{g}$, where g∈{1,2,4,8,32,128,512}.

We run control experiments 100 times for each configuration (l,n,g), with sentences randomly sampled from the dataset to reduce variance.

##### Counterbalanced Label assignment.

Assume we have a group A of sentences and a group B of sentences. To control for potential confounding factors arising from the LLMs’ response to labels (but not to sentence-label associations), we use a 2-by-2 experiment design: (i) assign labels (0, 1) to (A, B) and task the model to imitate label 0; (ii) assign labels (1, 0) to (A, B) and task the model to imitate label 0; (iii) assign labels (0, 1) to (A, B) and task the model to imitate label 1; (iv) assign labels (1, 0) to (A, B) and task the model to imitate label 1. The conditions (i) and (iv) are imitating group A sentences, and the conditions (ii) and (iii) are imitating group B sentences.

#### A.3.5 Quantifying control effect

Following the notations in Section A.3.1, we use $a_{i}^{l}\left[PC_{g};1\right]$ to denote the projection of neural activations onto a specific axis when prompted with $PC_{g}$ to imitate label 1 (similarly $a_{i}^{l}\left[PC_{g};0\right]$ for label 0).

We quantify the strength of neural control effects induced by control prompts $\left[PC_{g};0\right]$ and $\left[PC_{g};1\right]$ using Cohen’s d, which measures the standardized difference between two independent conditions (e.g., imitating label 0 vs. label 1). For each group of examples (of size $n_{1}$ and $n_{2}$), we compute:

$$d=\frac{{\bar{a}}_{i}^{l}\left[PC_{g};1\right]-{\bar{a}}_{i}^{l}\left[PC_{g};0\right]}{s_{\text{pooled}}},\;s_{\text{pooled}}=\sqrt{\frac{\left(n_{1}-1\right)s_{1}^{2}+\left(n_{2}-1\right)s_{2}^{2}}{n_{1}+n_{2}-2}},$$

where $\bar{.}$ denote the sample means and $s_{1}^{2}$, $s_{2}^{2}$ are the unbiased sample variances.

To estimate uncertainty, we compute the standard error of d using:

$$\text{S}{\text{E}}_{d}=\sqrt{\frac{n_{1}+n_{2}}{n_{1}n_{2}}+\frac{d^{2}}{2\left(n_{1}+n_{2}\right)}}.$$

Confidence intervals are reported as symmetric boundaries around d, i.e., $d\pm 1.96\times \text{S}{\text{E}}_{d}$.

### A.4 Additional results

#### A.4.1 Control Precision

We examine how precisely LLMs can modulate their internal representations along a specific neural direction (principal axis $PC_{g}$) as targeted by the prompts. Following the notations in Section A.3.1 and A.3.5, to assess whether this control effect aligns with the target axis or also influences other axes, we compute the absolute value of control effect $\left|d_{k}\left[PC_{g}\right]\right|$ of prompts $\left[PC_{g}\right]$ (g indexes the target axis) on PC axis k. The *target effect* is given by $\left|d_{g}\left[PC_{g}\right]\right|$. The average *target effect* is the mean value of axes: $\frac{1}{K}\sum_{k=1}^{512}\left|d_{k}\left[PC_{g}\right]\right|$

We define **control precision** as the ratio between these two quantities:

$$\text{ControlPrecision}\left(PC_{g}\right)=\frac{\left|d_{g}\left[PC_{g}\right]\right|}{\frac{1}{K}\sum_{k}\left|d_{k}\left[PC_{g}\right]\right|}.$$

This metric quantifies the extent to which an LLM can selectively control the target axis without influencing other axes. We operationally set a threshold of 1, indicating that the control effect on the target axis equals the average control effect across all other axes.

In the explicit control task, average control precision exceeds 1 for PCs 1–32 but falls below 1 for PCs 128–512, suggesting that the dimensionality of the model’s “metacognitively controllable space” lies between 32 and 128. This pattern is replicated in LLaMA-3.1 70B (Fig. A.2b).

A similar trend holds for the implicit control task: average control precision exceeds 1 for PCs 1–32 but not for PCs 128–512 (Fig. A.2a). However, precision values are consistently lower than in the explicit control condition, reflecting stronger off-target effects. This pattern is also replicated in the 70B model (Fig. A.2b).

#### A.4.3 Reporting performance of an ideal observer

Here, we aim to understand the theoretical upper bound of the reporting performance of LLMs. For an ideal observer, it has full access to all the neural activations of the LLM, serving as a theoretical upper bound of the reporting performance. Given a neural-defined label (either from a PC axis or LR axis), the optimal prediction can be achieved with a linear classifier (logistic regression). We analyze its reporting performance for each target PC axis and each model (Fig. A.5), which is much higher than the empirical reporting performance of LLMs (e.g., comparing the performance for llama 3.1 8B with Fig. 2c).
